# Purine Nucleotide Precursors in Preventing Myocardial Ischemia–Reperfusion Injury

**DOI:** 10.3390/ijms262110455

**Published:** 2025-10-28

**Authors:** Pawel Tomasz Musial, Piotr Arkadiusz Badtke, Magdalena Agnieszka Zabielska-Kaczorowska

**Affiliations:** Department of Physiology, Medical University of Gdansk, 80-210 Gdansk, Poland; pawel.musial@gumed.edu.pl (P.T.M.); piotr.badtke@gumed.edu.pl (P.A.B.)

**Keywords:** myocardial ischemia–reperfusion injury, nucleotides, purine metabolism, purine nucleotide precursors, D-ribose, AICAR, inosine, adenine, hypoxanthine

## Abstract

Changes in the homeostatic balance between purine nucleotide synthesis, degradation, and salvage are caused by disruptions in ATP supply and/or demand in the heart. These disruptions may affect myocardial energetics and, consequently, cardiac function and mechanics. Increased cardiac inorganic phosphate levels and decreased myocardial ATP levels are the outcomes of this decrease in purine nucleotide levels. Both modifications can immediately affect cellular mechanical work and tension development. Depletion of cardiac nucleotides and compromised myocardial mechanical function are linked to both acute myocardial ischemia and decompensatory remodelling of the myocardium in heart failure. Theoretically, in both acute ischemia and chronic high-demand situations associated with the development of heart failure, an imbalance in the breakdown, salvage, and synthesis of purine nucleotides results in a net loss of purine nucleotides. It was found that the use of nucleotide precursors can be a potentially effective approach to diminishing ischemia–reperfusion damage. The scope of this article is to review knowledge of the effect of purine nucleotide precursors such as D-ribose, AICAR, inosine, hypoxanthine, and adenine on myocardial ischemia–reperfusion injury and highlight potential targets for treating myocardial metabolic and mechanical dysfunction associated with ischemia–reperfusion injury by these molecules.

## 1. Introduction

**Figure 1:**
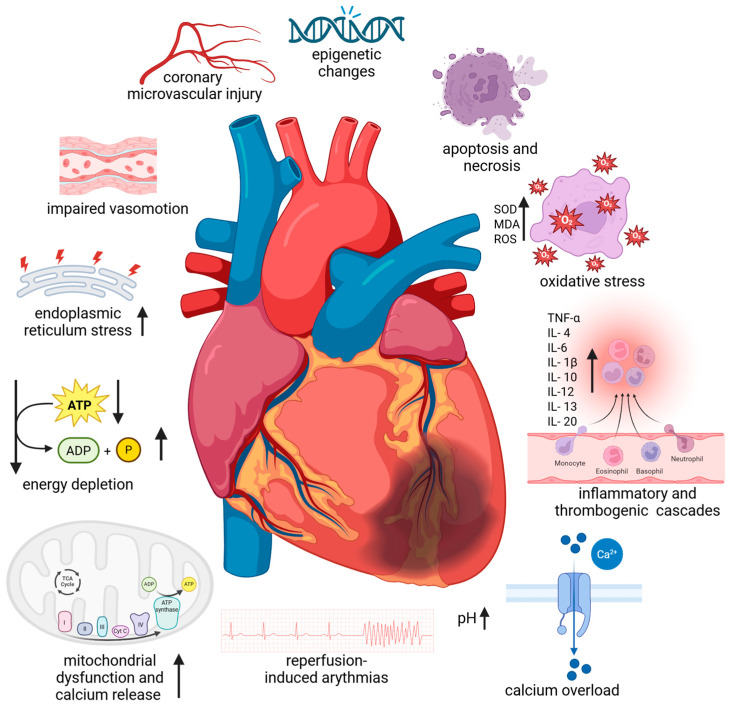
Pathophysiological mechanism of myocardial ischemia–reperfusion injury. Abbreviations: ATP—adenosine triphosphate; ADP—adenosine diphosphate; IL—interleukin; MDA—malondialdehyde, ROS—reactive oxygen species; SOD—superoxide dismutase, TNF-α—tumour necrosis factor-α; ↑—increase; ↓—decrease. Created in BioRender. Anielska, A. (2025) https://BioRender.com/xxktyf4. “(accessed on 24 October 2025)”.

Coronary heart disease (CHD) is one of the most common causes of morbidity and mortality worldwide [1]. The term myocardial ‘ischemia–reperfusion injury’ (IRI) is closely associated with CHD. Despite its crucial importance for cardiac cell survival, reperfusion initiates a wide and complex range of responses in the body (Figure 1) [2]. These responses can both exacerbate local damage and induce impairment of general body functions. Conditions leading to CHD include various forms of acute vascular occlusion (stroke, myocardial infarction, limb ischaemia) along with appropriate reperfusion strategies (thrombolytic therapy, angioplasty, surgical revascularisation). Routine surgery, organ transplantation, tissue transfer, cardiopulmonary bypass, vascular surgery, and trauma/shock may also be associated with tissue IRI [3]. Over the past few decades, substantial research has been conducted on the mechanisms that control necrosis and apoptosis, as well as the extensive investigation of cardioprotective processes [4].

It is known that rapid intracellular sodium, hydrogen, and calcium ion buildup brought on by ischaemia results in tissue acidosis. Adenosine triphosphate depletion, myofibrillar hypercontractility, mitochondrial ultrastructural destruction, and myocardial stunning are the outcomes [5]. The function of the mitochondria as regulators of energetics and cell viability has been the subject of research on cardioprotection and cell death [5,6]. Due to their high energy requirements, cardiac myocytes have a high mitochondrial density. IRI damage is one of the pathogenic situations that can cause mitochondria to degrade. Large amounts of reactive oxygen species (ROS) and proapoptotic signals are produced by these organelles, and they are essential for the start and continuation of the inflammatory phase of IRI. Due to energy stress and the overproduction of harmful reactive oxygen species, mitochondrial injury can be a major contributor to cardiac injury. This can result in oxidative stress, increased calcium release, and necrotic and apoptotic cell death. Furthermore, circulating leukocytes and cardiac resident macrophages also contribute to the inflammatory response. Leukocytes can readily penetrate the interstitial tissue through the ischaemic-damaged vascular endothelium. Cell necrosis and, to a lesser extent, apoptosis and autophagic processes trigger the activation of parenchymal and cardiomyocyte cell death programs if the ischaemic phase is prolonged enough. Restoring blood flow may exacerbate tissue injury by triggering the complement system and producing more ROS after an abrupt reoxygenation [5].

One important limiting factor in the recovery of myocardial ATP following an ischaemic insult is the availability of myocardial precursors [7]. At the site of cell injury, nucleotides are created and released, which can have both calming and upsetting effects. By restoring ATP levels, the main energy molecule in cells, supplements of these precursors can help shield heart tissue from damage. These substances may lessen cell death, enhance cardiac function, and improve outcomes following ischaemic episodes by improving the heart’s capacity to regain its energy balance [8]. This review is focused on human purine nucleotide precursors, such as D-ribose, AICAR, inosine, hypoxanthine, and adenine, in the prevention of ischemia–reperfusion injury rather than giving a general overview of the numerous aspects of purine biochemistry.

## 2. Human Purine Metabolism

**Figure 2:**
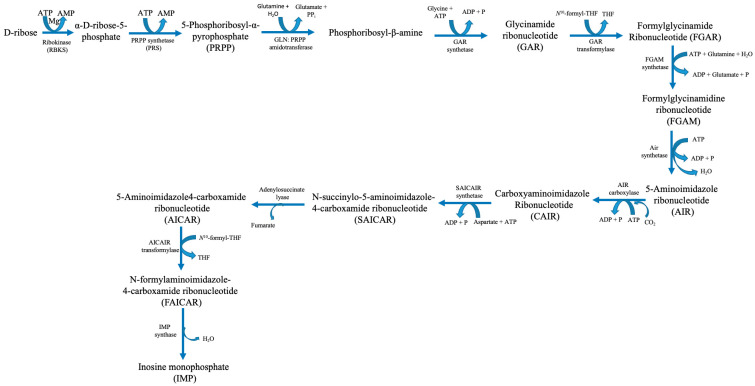
Human purine synthesis pathway. Abbreviations: ATP—adenosine triphosphate; ADP—adenosine diphosphate; AMP—adenosine monophosphate; H_2_O—water; Mg—magnesium; P—phosphate; PPi—pyrophosphate; THF—tetrahydrofuran.

The goal of purine metabolism in humans is to maintain the quantity of nucleotides in the tissues at an ideal level. Nucleotides are involved in the construction of DNA and RNA, energy metabolism, and the regulation of multiple metabolic pathways through the allosteric effects of enzymes or adenylate energy charge. Purine metabolism consists of de novo synthesis, salvage pathways, and catabolism. The mechanisms involved in purine metabolism include the interconversion of nucleotides and the breakdown of excess nucleotides. De novo and salvage syntheses, as well as the interconversion between AMP and GMP, are regulated by nucleotides. Nucleotides can be synthesised from tiny non-purine molecules via an energy-intensive de novo pathway or from existing purines via cost-effective salvage pathways. Phosphoribosyl pyrophosphate (PRPP), the primary precursor for de novo purine biosynthesis, is produced by the highly controlled enzyme PRPP synthetase (PRS), which requires ATP and ribose-5-phosphate from the pentose phosphate pathway as substrates (Figure 2) [5].

Six enzymes catalyse ten consecutive enzymatic processes that result in the assembly of a purine base onto a PRPP backbone. These enzymes interact within cells to form multi-enzyme complexes known as purinosomes. This process is known as “de novo purine biosynthesis” [6]. PRS catalyses the production of PRPP, an essential building block for purine biosynthesis. Moreover, it also plays a role in pyrimidine synthesis and the purine salvage pathways. IMP catalyses two successive enzymatic processes that produce GMP and AMP. Following phosphorylation, these mononucleotides yield nucleoside di- and tri-phosphates GDP, GTP, ADP, and ATP in that order. ITPase breaks down phosphorylation and deamination, producing by-products called XTP and ITP [5]. ATP, a high-energy phosphate molecule, is the main fuel used by the heart muscle, the essential engine for blood circulation, to carry out contraction and relaxation for blood pumping. ATP hydrolysis is the thermochemical engine that powers biosynthetic pathways, cellular signalling, and ion homeostasis. The powerhouse cellular organelles, mitochondria, produce the vast majority of ATP (80%) in cardiac cells via aerobic oxidative phosphorylation in the electron transport chain. Human cardiac cells contain a large number of mitochondria, which make up approximately 40–50% of the cardiac cellular mass, to manufacture enormous quantities of ATP to sustain the functional demands of the heart [8]. Under basal conditions, the human heart resynthesizes its entire ATP pool multiple times per minute by continual hydrolysis and synthesis [8].

## 3. Human Purine Metabolism in Cardiac Ischemia

**Figure 3:**
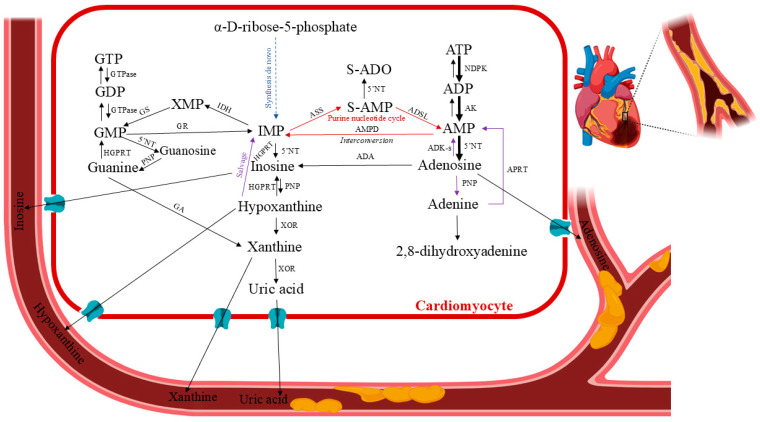
Human purine metabolism in cardiac ischemia. Pertinent pathways: formation of the purine nucleotide inosine monophosphate (IMP) from non-purine precursors (synthesis de novo) or purine bases (salvage reactions); purine nucleotide interconversion reactions; degradation to the end product uric acid (catabolic reactions). Abbreviations: 5′NT—5′-nucleotidase; ADA—adenosine deaminase; ADK-s—adenosine kinase short isoform; AK—adenosine kinase; ADP—adenosine diphosphate; AMP—adenosine monophosphate (or adenylic acid); AMPD—AMP deaminase; ASS—adenylosuccinate synthetase; ATP—adenosine triphosphate; GA—guanase; GDP—guanosine diphosphate; GMP—guanosine monophosphate; GR—GMP reductase; GS—GMP synthase; GTP—guanosine triphosphate HGPRT—hypoxanthine–guanine phosphoribosyltransferase; IDH—IMP dehydrogenase; IMP—inosine monophosphate; NDPK—nucleoside-diphosphate kinase; PNP—purine nucleoside phosphorylase; S-AMP—succinyl-AMP or adenylosuccinate; XOR—xanthine oxidoreductase. Created in BioRender. Anielska, A. (2025) https://BioRender.com/p2ftrx0. “(accessed on 16 May 2025)”.

Myocardial ischemia results in an inadequate blood supply to the heart tissue, leading to morphological and functional abnormalities. It develops when the heart’s fractional consumption of oxygen is inadequate to sustain the rate of cellular oxidation. This oxidative stress can lead to necrosis and apoptosis in cardiomyocytes, as well as damage to cellular proteins. Moreover, reduced local perfusion causes severe dysfunctional changes, such as myocardial stunning, myocyte death, reperfusion arrhythmia, oedema, and numerous other consequences stemming from a vicious loop of vascular, endothelial, and mitochondrial malfunctions [9]. Clinical and experimental evidence indicate that the major processes leading to tissue and cell dysfunction are primarily associated with reperfusion [10]. To accommodate chronic low myocardial hibernation and preconditioning, the ischemic tissue and the rest of the body may undergo biochemical and molecular adaptive modifications. Chronic ischemia develops gradually and silently over months or years. It damages most of the body’s organs. The pathophysiology of ischemia–reperfusion injury has been extensively studied over the past 40 years, and current therapeutic strategies attempt to incorporate a wide range of factors, including the impairment of endothelial relaxation following ischemia–reperfusion, reduction of reperfusion injury through the scavenging of free radicals, and inhibition of neutrophil adhesion and activation [11,12]. Due to the high oxygen dependence of this vital aerobic process, there are significant metabolic ramifications from coronary circulation disruption or reduction, which lowers the oxygen supply to the affected area of the heart (ischemia), as well as from other forms of hypoxia (a lack of oxygen in the heart tissues) caused by environmental factors, demand/supply imbalances, etc. During episodes of restricted blood flow, like myocardial infarction, the heart’s energy stores are depleted, leading to tissue damage [7]. After a brief period of ischemia, the heart can only slowly replenish its adenine nucleotide pool. It was found that normal ATP levels were not restored until several days after reperfusion [13]. This phenomenon, functionally known as stunned myocardium, is caused by the ability of ATP degradation products produced during ischemia to permeate the cell membrane (Figure 3). They are removed from the heart and washed out, making them unavailable for instant intracardiac reutilization through salvage pathways. ATP and phosphocreatine are rapidly depleted at the onset of myocardial ischemia/hypoxia. After 15 min of total ischemia, the heart tissues would lose 65% of their ATP levels [14]. Adenosine diphosphate (ADP) and adenosine monophosphate (AMP) are two ATP catabolic byproducts produced during ischemic events. Additionally, these events activate enzymes that are normally dormant, such as purine nucleoside phosphorylase (PNP), adenosine deaminase (ADA), 5′-nucleotidase (5′NT), and xanthine oxidoreductase (XOR), which catabolize AMP into adenosine, inosine, hypoxanthine, xanthine, and uric acid [15,16,17,18,19,20,21]. After ischemia, ATP levels are restored by the adenine nucleotide de novo synthesis pathway, which depends on the availability of phosphoribosyl pyrophosphate, and the purine salvage system, which depends on adenosine, adenine, and hypoxanthine as substrate precursors [22,23,24,25,26]. Consequently, ischemia–reperfusion injury research must be up-to-date with the status of drug protection so that translational research efforts can be optimised to turn this information into novel human therapies [27].

## 4. Purine Nucleotide Precursors Prevent Ischemia/Reperfusion Injury

### 4.1. D-Ribose

Supplementing with D-ribose has been shown in multiple animal experiments to significantly improve function and enhance the restoration of ATP levels after global and regional myocardial ischemia (Table 1). D-ribose bypassing the hexose monophosphate shunt is immediately metabolised to ribose 5-phosphate, thus increasing the available pool of PRPP, an essential precursor substrate for the synthesis of adenine nucleotides. By increasing PRPP, whose synthesis is the rate-limiting stage in the de novo synthesis of adenine nucleotides, D-ribose raises ATP levels and improves cardiac function [28]. According to Zimmer and Gerlach, adenine nucleotide synthesis was found to rise in adult, isolated rat hearts upon supplementation with D-ribose [29]. Mitochondria control numerous metabolic and signalling processes; however, their principal function is ATP synthesis. Compromised mitochondrial activity can lead to diminished cellular respiration efficiency and a consequent reduction in the production of ATP. D-ribose is a naturally occurring ATP substrate found within cells. Supplemental D-ribose has demonstrated efficacy in facilitating the recovery of lowered nucleotides. Consequently, D-ribose supplementation may facilitate the restoration of adenine nucleotides within the cell, potentially serving as a therapeutic alternative for many pathophysiological diseases [30].

In isolated working rat hearts, administration of D-ribose to cardiac perfusion before and after ischemia enhanced post-ischemic myocardial ATP levels and functional recovery of the heart. There are a few possible mechanisms responsible for this ribose-enhanced recovery, such as adenine nucleotide degradation during ischemia, pathways involved in de novo and salvage adenine nucleotide synthesis, manipulation of the rate-limiting steps of these synthetic pathways, and, ultimately, the effect these pathways have on post-ischemic myocardial ATP and functional recovery [17]. It was found that the rates of hypoxanthine and adenine were higher in cultured myocytes and the myocardium of rats administered with D-ribose [22,31]. After 20 min of global ischemia, St. Cyr et al. noted that supplementation with D-ribose and adenine led to an 85% recovery in ATP levels, when there was no ATP recovery in the absence of D-ribose and adenine [32]. Schneider et al., in the same chronic canine model but with additional functional instrumentation, reported similar ATP benefits with D-ribose and adenine, as well as improvements in left ventricular non-compliance/diastolic dysfunction following 20 min of global myocardial ischemia [33,34]. Tveter et al. examined the effects of D-ribose given alone after subjecting the hearts to a moderate global myocardial ischemia insult lasting 20 min. They discovered that, as previously noted with D-ribose and adenine, D-ribose alone produced comparable advantages in both myocardial energy metabolite levels and functional recovery following global ischemia [35]. D-ribose seemed to ameliorate the adenine nucleotide pool after ischemia, diastolic dysfunction, and the recovery of myocardial adenine nucleotide levels [36]. After myocardial infarction, Zimmer et al. observed a decrease in left ventricular hemodynamics in adult rats. Following the infarction, they observed decreased cardiac output and stroke volume indices, a progressive reduction in left ventricular systolic pressure, a decline in left ventricular dP/dtmax, and higher left ventricular end-diastolic pressure. The above-measured left ventricular hemodynamic parameters improved with stimulation of adenine nucleotide synthesis upon the supply of the substrate D-ribose [37]. Similarly, Befera et al. found that supplementing adult rats with D-ribose after an acute myocardial infarction produced comparable results. When D-ribose was supplied, they observed an improvement in the left ventricular function in the remote left ventricular zones. When D-ribose supplementation was administered, ventricular dilatation decreased, and contractility and cardiac wall thickness increased [38]. D-ribose was shown to either prevent or postpone the onset of left ventricular dysfunction that followed an acute myocardial infarction in two preclinical animal experiments. Gonzalez et al. found that administering adult rats before infarction significantly reduced the extent of the generated left ventricular infarct and significantly improved left ventricular function when measured six hours after the infarct. With additional D-ribose, left ventricular relaxation measures were significantly improved, and left ventricular systolic pressure and contractility returned to normal [39]. In rats, continuous intravenous infusion of D-ribose during recovery from 15 min of myocardial ischemia leads to the restoration of the cardiac ATP pool within 12 h. Without any intervention, 72 h were needed for ATP normalisation [40]. Pre-treatment with bolus intravenous D-ribose markedly enhanced tolerance to global ischemia in normal rat hearts, which is linked to lower rates of high-energy phosphate consumption. LV function at baseline was greatly enhanced in hypertrophied hearts using this pretreatment strategy. However, it did not affect the baseline metabolites or the heart’s tolerance to global ischemia [41]. Interest in D-ribose supplementation as a potential diagnostic and therapeutic agent for patients with ischemic cardiovascular diseases was sparked by the promising results of pre-clinical animal studies. Its potential benefits in patients with ischemic congestive heart failure, during and after cardiovascular surgery, and its diagnostic potential in recognising myocardial hibernation, which is present in ischemic coronary artery disease, have also been noted [42]. Owing to the advantages of D-ribose during and after ischemia, scientists have investigated its potential to improve the detection of hibernating cardiac fragments. The term “hibernating myocardium” refers to localised myocardial dysfunction caused by extended ischemia or hypoperfusion. When sufficient blood flow is restored to these areas, there is a possibility of restoring the tissue’s functionality, and an increase in cardiac ATP levels can occur [43]. Currently used techniques for locating these areas include magnetic resonance imaging, dobutamine stress echocardiography, PET, and Thallium-201 scans [36]. Preclinical animal research showed the benefits of D-ribose in the hibernating myocardium before conducting clinical trials. Perlmutter et al. used Thallium-201 scans to identify more reversible abnormalities in the clinical setting. D-ribose improved the identification of the ischemic myocardium and appeared to facilitate 201Tl redistribution in CAD (coronary artery disease) patients [44]. D-ribose also improved SPECT Thallium imaging detection of viable ischemic cardiac areas. In patients with CAD, D-ribose improved the identification of viable ischemic myocardium by enhancing the detection of thallium redistribution at 4 h compared to 24-h control pictures [45]. Recently, Sawada et al. showed that adding D-ribose to a dobutamine stress echocardiogram improved the detection of abnormalities related to wall motion dysfunction while also having anti-ischemic benefits [46]. Another study was conducted which individuals with stable coronary artery disease regularly completed treadmill exercise testing while taking D-ribose supplements.

**Table 1:** A table summarizing key studies relevant to purine nucleotide precursors in preventing myocardial ischemia–reperfusion injury.

Purine Nucleotide Precursor	Objectives	Significant Findings	Implications for Future Research	Study References
D-ribose	Isoproterenol-induced alterations in cardiac adenine nucleotides and morphology by D-ribose.	Supplementation with D-ribose decreased the incidence of isoproterenol-induced cardiac cell damage, and the diminution of adenine nucleotides was completely avoided.	Heart necrosis develops as a result of adenine nucleotide deficiency.	Zimmer, H.-G.; 1980 [28]
	Determination of the effects of D-ribose infusion in a long-term model of global ischemia.	D-ribose infusion significantly enhanced the recovery of energy levels in the postischemic heart.	D-ribose could be a promising therapeutic agent for enhancing cardiac function after ischaemic events. Further research is necessary to investigate the underlying mechanisms	St. Cyr, J.A.; 1989 [32]
	A study of the effect of D-ribose on heart function and infarct size after myocardial infarction (MI).	Six hours after MI, ribose treatment dramatically decreased MI size and enhanced left ventricular function. Ribose treatment contributes to maintaining the remote myocardium’s function	Increasing myocardial energy levels enhances function and may postpone long-term alterations, such as apoptosis, in several surgically curable chronic heart failure disorders.	González, G.E.; 2009 [39]
	D-ribose supplementation for patients with congestive heart failure (CHF) who often reported exhaustion and dyspnoea.	Patients with class II–III CHF and left ventricular dysfunction were able to maintain their VO_2max_, and they increased their ventilatory efficiency. They showed a satisfactory trend in their daily quality of life assessment when they supplemented with D-ribose.	For advanced CHF, D-ribose should definitely be taken into consideration as an addition to standard therapy regimens.	Carter O.; 2005 [47]
	Determination of whether ubiquinol and/or d-ribose would reduce the symptoms and improve cardiac performance in patients with heart failure with preserved ejection fraction (HFpEF).	A treatment with ubiquinol or D-ribose improved EF and production of ATP while lowering HF symptoms in patients with HFpEF. The results were not further improved by adding D-ribose to ubiquinol treatment, indicating that either supplement, by itself, is adequate to enhance physiologic variables and symptoms, but requires the study dosage.	Phase 3 clinical studies, which are carried out in numerous clinics worldwide, must involve a greater number of patients.	Pierce, J.D.; 2020 [48]
AICAR	Investigation of the acute effects of AICAR on adenine nucleotides, inosine monophosphate (IMP), and postischemic ventricular function	The rise in IMP indicates that AICAR was phosphorylated and incorporated in the normal and postischemic myocardium over a comparatively brief perfusion interval, but it was unable to improve function recovery or raise AMP or ATP levels.	AICAR was not a useful pharmacologic technique for evaluating the connection between the recovery of ventricular function and the postischemic adenine nucleotide pool.	Mentzer, R.M.; 1988 [49]
	Evaluation of the effects of AICAR on myocardial ischemia, left ventricular function, myocardial infarction, heart failure, life-threatening dysrhythmias, and death in patients undergoing coronary artery bypass graft (CABG) surgery.	The study demonstrated that the administration of AICAR perioperatively is safe in patients undergoing CABG surgery. It limits the severity of post-bypass myocardial ischemia as shown by shorter ischemic duration in patients receiving high doses of AICAR.	More research was needed to evaluate whether AICAR can significantly reduce the incidence of myocardial infarction and the severity of cardiac damage.	Leung, J.M.; 1994 [50]
	Involvement in improving contractile dysfunction by AICAR by increasing adenosine release in ischemic myocardium.	The results indicate that reperfusion injury is unavoidable but can be mitigated. The study demonstrated that AICAR administration significantly enhanced contractile dysfunction after a short duration of myocardial ischemia through adenosine-dependent mechanisms.	Additional efforts are required for the clinical application of AICAR.	Kitakaze, M.; 1999 [51]
	AICAR-dependent AMPK activation involvement in improving scar formation in the aged heart in a model of MI	AICAR avoids unfavourable remodelling and enhances post-ischemic cardiac function.	A new treatment approach for preventing harmful remodelling in the ageing heart may result from these findings.	Cieslik, K.A.; 2013 [52]
Inosine	Regulatory mechanisms involved in the therapeutic use of purines for the treatment of ischemic heart disease.	Inosine and hypoxanthine were incorporated into both the ATP and GTP pools in the heart. This process is stimulated after ischemia and by ribose perfusion and is thereby dependent on myocardial PRPP concentrations.	Inosine seemed to restore ATP levels, which could be beneficial after ischemia	Harmsen, E.; 1984 [31]
	The influence of supplementation with inosine of cold cardioplegia (CPS) and recovery perfusate on the cardiac output, ATP and total adenine nucleotide content.	Nucleotide levels and cardiac output recovery were enhanced by the addition of inosine to the recovery perfusate and CPS.	These findings suggest that functional recovery from cardioplegia is hindered by the washout of nucleotide breakdown products in the cytosol or during reperfusion, which prevents their rescue for nucleotide resynthesis.	DeWitt, D.F.; 1983 [53]
	The effects of inosine on ischemia/reperfusion injury in a rat heart transplantation model.	Inosine enhanced myocardial and endothelial function at early reperfusion after heart transplantation with a continuing protective effect against reperfusion-induced graft coronary endothelial dysfunction. Peroxynitrite-poly(ADP-ribose) polymerase (PARP) pathway modification may be at least partially responsible for inosine’s actions.	Inosine seemed to function as a nonprofessional but rather effective inhibitor of PARP activation. To clarify the precise mechanism of action of inosine therapy, more research is required.	Szabó, G.; 2006 [54]
	Plasma inosine levels as a valuable diagnostic marker of pre-necrosis cardiac ischaemia.	A possible biomarker for early cardiac ischaemia could be the amount of inosine present in animals exposed to cardiac oxidative stress.	Increased inosine levels could be indicative of early cardiac ischaemia and should be determined by preliminary human investigations.	Farthing, D.; 2006 [55]
Adenine	The effect of adenine on myocardial ATP content in the post-anoxic nonworking rat heart	The results demonstrated that a 50 μM dose of adenine could regulate ATP concentrations during 60 min of anoxia in the nonworking rat heart; however, increasing the adenine dosage to 1 mM resulted in a decrease in tissue ATP concentration.	Data suggested a potential dose-dependent effect of adenine on ATP metabolism, highlighting the need for further investigation into the mechanisms underlying these changes.	Halle, A.A.; 1989 [56]
	Development of concurrent kidney and cardiovascular injury induced by chronic dietary adenine intake.	Treatment with 0.25% adenine in rats resulted in chronic renal and cardiovascular damage. Cardiovascular alterations encompassed heightened ventricular fibrosis, elevated systolic blood pressure, increased left ventricular stiffness, and compromised vascular responses.	These findings suggest a significant correlation between adenine exposure and the deterioration of both renal and cardiovascular functions. Further investigation was warranted to elucidate the underlying mechanisms contributing to these adverse effects.	Diwan, V.; 2013 [57]
	Endogenous adenine as a potential driver of the cardiovascular-kidney-metabolic (CKM) syndrome	Research has demonstrated that endogenous adenine has a causative role in heart failure and ischemic heart disease within the context of CKM syndrome	The importance of further exploring the biochemical pathways involved in adenine metabolism. Understanding these mechanisms could lead to novel therapeutic strategies for managing heart-related conditions associated with CKM syndrome.	Tamayo, I.; 2024 [58]
Hypoxanthine	The development of the high-pressure liquid chromatographic system for the determination of purine nucleosides in the blood.	Increased ischemic heart’s synthesis of hypoxanthine was detected.	The high-pressure liquid chromatographic assay of blood hypoxanthine as a useful tool in the diagnosis of ischemic heart disease.	Harmsen, E.; 1981 [59]
	The development of a rapid and simple chemiluminescence method was developed for screening levels of inosine and hypoxanthine in human plasma	The capacity to distinguish among total hypoxanthine levels in healthy individuals and patients presenting with non-traumatic chest pain and possible acute cardiac ischaemia was proven by the fast chemiluminescence approach.	Chemiluminescence technology may be used as a diagnostic tool to quickly check for high levels of hypoxanthine and inosine in human plasma, which may be indicators of acute myocardial ischaemia.	Farthing, D.E.; 2011 [60]

D-ribose showed notable advantages in prolonging treadmill activity duration before the commencement of angina and/or ischemic electrocardiographic alterations [61]. In another study, patients with congestive heart failure (CHF) who often reported exhaustion and dyspnoea were investigated. As their condition becomes worse, they show a decline in ventilatory efficiency. Carter et al. discovered that patients with class II-III CHF and left ventricular dysfunction were able to maintain their VO_2max_. They increased their ventilatory efficiency and showed a satisfactory trend in their daily quality of life assessment when they supplemented with D-ribose [47]. In a different clinical study, Vijay et al. showed that D-ribose had beneficial effects on individuals with class II–IV CHF. Although it was not statistically significant, they noticed a good trend in class II patients’ improved ventilator efficiency. Moreover, they observed significant improvements in ventilatory efficiency in class III–IV CHF patients [62]. Additionally, the application of D-ribose in cardiovascular surgery has been investigated. Following surgery, changes in myocardial function during the post-ischemic interval were observed. According to Wyatt et al., using a cardioplegic solution comprising D-ribose, hypoxanthine, and adenosine during intraoperative ischemia preserved myocardial energy levels and improved functional recovery after surgery [63]. In contrast to the decline in ejection fraction seen in patients not receiving D-ribose, Vance et al. reported that parenteral use of D-ribose during elective aortic valve replacement, with or without concurrent coronary artery bypass grafting, resulted in the maintenance of ejection fraction postoperatively. They concluded that the D-ribose therapy plan preserved left ventricular function during the surgery [64]. The next experimental step involved oral D-ribose dietary supplementation. In clinical studies, 5 g/dose of D-ribose supplementation was used multiple times per day. Patients undergoing off-pump coronary artery bypass revascularisation treatment were administered oral D-ribose as part of a metabolic-tailored protocol. Perkowski et al. discovered that in patients undergoing “off-pump” coronary artery revascularisation, the D-ribose metabolic strategy led to a significant early postoperative improvement in the cardiac index, as well as decreased mortality and morbidity. Supplementation with D-ribose was linked to an improvement in ventricular systolic function as measured by the cardiac index, even though the D-ribose group showed signs of a higher total atherosclerotic burden, more advanced comorbidities, and trends toward a higher average age [65]. Omran et al. investigated the usefulness of D-ribose in patients with class II–III CHF due to its capacity to reduce diastolic dysfunction and accelerate the recovery of cardiac ATP levels after ischemia. They mentioned the objective and subjective advantages of D-ribose. Repeated echocardiography examinations revealed a decrease in diastolic dysfunction parameters. In addition, patients reported improved subjective quality of life and physical function [66]. Researchers observed an improvement with ribose in a group of patients with advanced heart failure who were undergoing ventilatory exercise. Along with a 44% improvement in Weber class, all patients showed significant improvement in ventilatory parameters at the anaerobic threshold [67]. In another set of experiments, it was investigated whether ribose could make individuals with coronary artery disease (CAD) more tolerant to myocardial ischemia. Twenty men with confirmed severe CAD underwent two symptom-limited treadmill activity tests on back-to-back days. It was hypothesised that the resulting ischemia may alter ATP metabolism in a way that would continue for several days. The authors concluded that oral ribose therapy for three days increased the heart’s tolerance to ischaemia caused by exercise testing in patients with CAD [61]. Recently, several studies have discussed the use of D-ribose as a therapeutic agent in patients with heart failure with preserved ejection fraction (HFpEF). Patients with HFpEF may experience sensations of exhaustion and dyspnea. Data about HFpEF is linked to compromised myocardial bioenergetics. Implementing D-ribose enhanced mitochondrial function by elevating ATP levels and improving cardiac performance in those patients. A recently concluded clinical trial involving HFpEF patients demonstrated that D-ribose enhanced ATP generation and improved cardiac ejection fraction [68]. By administering oral D-ribose, patients with HFpEF obtain an additional substrate to bypass the glucose-6-phosphate dehydrogenase step and possibly raise their myocardial ATP levels [48]. A 12-week course of ubiquinol (600 mg daily) or D-ribose (15 g daily) reduced heart failure symptoms, BNP, and lactate levels in patients with HFpEF, while increasing ejection fraction and ATP production [69]. In summary, for individuals with cardiac ischemic injury or HFpEF additional D-ribose may serve as a helpful therapeutic option. D-ribose is believed to support energy production in cardiomyocytes through mitochondrial bioenergetic pathways, potentially improving heart function and reducing symptoms [68].

### 4.2. 5-Aminoimidazole-4-Carboxamide Ribonucleotide (AICAR)

5-Aminoimidazole-4-carboxamide ribonucleoside (AICAR) is a commonly used pharmacological modulator of cell metabolism [70]. It has been claimed that AICAR accelerates the rate of de novo cardiac ATP synthesis. The cardiac cell takes up this compound and phosphorylates it, allowing it to enter the de novo synthesis route to the more highly controlled regulatory sites. Additionally, AICAR can be phosphorylated in some cell types to produce AICA ribotide (ZMP), an AMP analogue that is capable of activating AMPK [71]. ZMP may also block AMP deaminase and enter the de novo pathway for adenosine synthesis. Both pathways may be involved in the increase in ATP and/or adenosine levels. There is disagreement, nonetheless, regarding whether the heart produces ZMP. While some research linked cardiac effects to AMP kinase activation, another study showed that the heart does not produce ZMP, in contrast to the liver [72,73]. In the ischemic or metabolically inhibited heart, the main actions of AICAR include blocking adenosine kinase and adenosine deaminase activities, which results in an increase in the amount of adenosine produced overall. AICAR, as an AMPK activator, has demonstrated the capacity to enhance mitochondrial biogenesis and fatty acid oxidation [74,75]. In the study by Du et al., AICAR preserved cardiomyocytes and mitochondria by activating AMPK signalling, which was contingent upon Drp1-mediated mitochondrial dynamics. The findings not only established a theoretical basis for investigating the mechanism but also proposed a possible therapeutic target for myocardial ischemia/reperfusion injury [76]. Initial investigations on AICAR showed that by raising ATP concentrations, it may shield the heart from ischemia [77]. For example, it was demonstrated that AICAR protected the rat heart against ischemic damage in acute ischemia/reperfusion and transplantation models with persistent ischemia [78]. Researchers measured how AICAR influenced the rate of myocardial adenosine triphosphate synthesis after coronary artery occlusion in the dog. The study measured a 20-fold increase in adenine nucleotide synthesis in non-ischemic tissue during de novo synthesis, which was assessed by [14C]glycine incorporation. Incorporation of tritium-labelled AICAR into ATP was demonstrated within the first 3 h of reperfusion in the study with more severe ischemia. Additionally, a reduction of ATP to 50% of normal concentration was noticed. Within three hours of reperfusion, this acceleration, which was twice as significant as that of D-ribose, was still too small to be picked up from the total amount of ATP in the tissue. In the experiment with AICAR infusion, no rise in the tissue inosine or hypoxanthine concentrations was found. There are two explanations for whether AICAR’s acceleration of ATP synthesis is insufficient. The first is that myocyte phosphorylation of AICAR to AICAR monophosphate is inefficient, especially in postischemic tissue. In the studies on the ischemic heart, AICAR concentrations were twice as high as AICAR monophosphate concentrations, and four times higher than in non-ischemic tissue. The second explanation is that the conversion of AICAR monophosphate to AICAR triphosphate is the preferred metabolic pathway at increasing concentrations of AICAR monophosphate [79]. In the other experiments, the acute effects of AICAR on adenine nucleotides, IMP, and postischemic ventricular function in an isolated rat heart preparation were investigated [49]. The dosage of 100 µmol/L AICAR was chosen for the study of Mentzer et al. Lower concentrations (10 to 50 µmol/L) used earlier did not increase the IMP level during perfusion intervals, nor did they increase the total adenine nucleotide pool. This study was consistent with a previous report that found accumulation of AICAR phosphates, which inhibited adenylosuccinate lyase, suggesting that the failure of AICAR to augment repletion of the postischemic adenine nucleotide pool is caused by the myocardium’s inability to convert IMP to AMP [80]. Adenylosuccinate lyase is necessary to metabolise IMP into adenylosuccinate, which is AMP’s precursor. As a result, although AICAR may accelerate the rate of de novo synthesis, it successfully inhibits the increase in ATP synthesis. Summarising, AICAR appeared not to be a useful pharmacological compound for evaluating the connection between the recovery of ventricular function and the postischemic adenine nucleotide pool [49]. In the other experiments, the enhancement of scar formation in the ageing heart by AICAR-dependent AMPK activation in a mouse model of ischemia/reperfusion injury was demonstrated. AMPK activation increased myofibroblast count in the infarcted area. Moreover, activated AMPK enhanced collagen maturation and improved scar formation in the heart. AICAR improved post-ischemic heart function and prevented adverse cardiac remodelling [52]. It was found by Moopanar et al. that AICAR at a dose of 500 μM inhibited cardiac sodium–hydrogen exchanger (NHE1). This inhibition greatly contributed to the cardioprotective effects of AICAR in the isolated rat heart after an ischemic period. AICAR was tested on humans at much lower concentrations (10–20 μM) as a cardioprotective drug, and it seems unclear whether the inhibition of NHE1 constitutes more than a modest part of the mechanism of action. Although the exact mechanism by which AICAR inhibits NHE1 is unknown, it does not seem to function via activating AMPK or releasing adenosine [81]. In the recent study of Du et al., using isolated mouse hearts in a Langendorff perfusion system, AMPK signalling, which was dependent on regulating GTPase dynamin-related protein 1 (Drp1)-mediated mitochondrial dynamics, was activated by AICAR to protect cardiomyocytes and mitochondria from ischemia/reperfusion injury [76]. According to research, cardiac injury during haemorrhagic shock appears to be age-dependent and is linked to AMPK activation, age-dependent cellular localisation, and deregulation of downstream metabolic pathways. Both young and adult mice’s cardiac damage was mitigated by AICAR treatment. Nevertheless, the drug’s age-dependent molecular mechanisms of action and cardioprotective effects in ischemic injury [82]. The use of AICAR before heart donation in rats resulted in a significant reduction in cardiac graft damage caused by cold ischaemia/reperfusion. It was suggested that the main ways in which this therapy protects the heart are by controlling heart energy metabolism, phosphorylating eNOS, lowering ROS levels, and preventing the opening of the mitochondrial permeability transition pore in the mitochondria [83].

Following the success of animal studies, numerous clinical trials examining the impact of AICAR on the risk of ischemic heart injury after coronary bypass grafting were conducted [50,84,85,86]. Although the small effects from individual trials were not statistically significant, a meta-analysis of five of these trials revealed that AICAR decreased the frequency of myocardial infarctions and the rate of early death following coronary bypass surgery [84,86]. Even though AICAR helped patients recover from ischemia, it is unclear exactly how it worked in humans. There is little evidence to support the early theory that higher ATP levels were the cause of the advantages [79,87]. Rather, it has been suggested that the advantages are secondary to adenosine’s cardioprotective properties and result from the increased synthesis of adenosine [88]. The adenosine receptor blocker 8-sulfophenyltheophylline (8-SPT) has been demonstrated to obstruct the protective effects of AICAR, which lends credence to this notion [89]. As demonstrated by Kitakaze et al., the advantageous effects of AICAR on adenosine release are mainly due to contractile dysfunction in the myocardium that has been stunned. Furthermore, the enhanced release of adenosine may be responsible for increases in ecto-5′-nucleotidase activity caused by AICAR. AICAR was reported to minimise ischemia and myocardial infarction during coronary artery surgery in humans, leading to better recuperation following surgery. The study demonstrated that AICAR administration markedly improved contractile function following a brief period of myocardial ischemia via adenosine-dependent mechanisms. Results suggested that reperfusion injury is unavoidable but can be mitigated [51]. The results of human trials showed that AICAR can lower the combined adverse cardiovascular events, myocardial infarction, and early cardiac death [70]. The RED-CABG experiment terminated in 2012, as preliminary results did not show a decrease in morbidity or death in patients at intermediate to high risk who received AICAR as compared to placebo [90].

### 4.3. Adenine

There are only a few reports about the influence of adenine supplementation on the cardiac tissue. It was found that cardiac muscle cells can use adenine as a substrate for ATP production. The absence of vasoactive characteristics of adenine makes it a potential therapeutic candidate as a high-energy precursor. A study published on the adenine dose effects on myocardial ATP level in the heart of a post-anoxic non-working rat showed that after 60 min of anoxia in the nonworking rat heart, adenine at a dosage of 50 μM brought ATP concentrations back to the normoxic range. On the other hand, increasing adenine to 1 mM decreased the tissue ATP concentration [56]. Adenine has been implicated in the development of ischemic heart disease and diabetic cardiomyopathy, according to experimental evidence [91]. We assume that further studies on adenine were dismissed due to elevated plasma uric acid levels, which may be the first cause of renal injury in rats administered adenine [91,92]. Being a purine base, adenine may be responsible for the formation of uric acid via activating xanthine oxidase, which may also be the cause of elevated oxidative stress, as seen by the increased expression of HO-1 in response to an adenine diet. Rats given a diet containing 0.25% adenine experienced renal and cardiovascular damage, which resulted in a decline in kidney and cardiovascular function analogous to that of human chronic kidney disease. Allopurinol corrected renal and cardiovascular alterations by lowering the elevated uric acid concentration, indicating that elevated uric acid production is the most likely pathogenic mechanism for kidney injury and related cardiovascular abnormalities [57]. The increase of adenine in the diabetic heart was identified in the current investigation of Tamayo et al. as a possible reason for the development of diabetic heart failure with HFpEF. The detrimental effect of adenine in the diabetic heart is believed to stem from a deficiency in mitophagy, the process responsible for the removal of damaged mitochondria [57]. Previous studies have associated human heart failure with impaired mitophagy and decreased bioenergetics [93]. Furthermore, increased production of adenine in heart tissue has been linked to an increase in cardiomyocyte size (hypertrophy) and a higher number of damaged mitochondria associated with enveloped mitochondria in the hearts of diabetic individuals. It was discovered that adenine accumulated in diabetic patients’ hearts and was linked to the level of renal function. Particularly, methylthioadenosine phosphorylase (MTAP) gene expression was elevated in endothelial cells in human hearts with hypertrophy, and adenine was elevated in coronary blood in both humans and diabetic mice [58]. Patients with diabetes who had higher urine adenine/creatinine ratio (UAdCR) levels were shown to be at higher risk of incident HFpEF. Because the reduction of adenine synthesis reduced the size of myocardial infarcts in a rat model and normalised the characteristics of diabetic cardiac dysfunction in a diabetic mouse model, endogenous adenine appeared to be in the causal pathway. Thus, endogenous adenine synthesis could be an essential compound that connects renal and metabolic failure to cardiovascular disease [58].

### 4.4. Inosine

The human body naturally produces inosine, an endogenous purine nucleoside, as a breakdown product of purine metabolism. With a half-life of <5 min, inosine is metabolised in red blood cells and present in trace amounts (e.g., low ng mL^−1^) in human plasma [94]. The salvage pathway, a cellular biochemical mechanism in humans that helps conserve energy in producing the large amounts of ATP needed for cardiac cellular use, can recycle cellular inosine by converting it back to ATP through some enzymatic steps. But during prolonged cardiac oxidative stress (e.g., 20 min), cardiac cells accumulate large amounts of ATP metabolic byproducts, which trigger normally inactive enzymes to catabolise ATP byproducts, making them available throughout the body before being eliminated. It is known that under hypoxic conditions and contrary to normoxic conditions, myocardial cells produce great quantities of both adenosine and inosine. Said briefly, during hypoxia, adenosine is utilized mainly for cell protection mechanisms, via stimulation of myocardial cell and endothelial cell adenosine A1, A2, and A3 receptors, leading to various biological effects, including activation of the mitochondrial K^+^ ATP-dependent channels. At the same time, inosine is used for cell energy repletion [55]. The fundamental paper published a half-century ago by the renowned cardiovascular physiologist Robert M. Berne in 1963 included the first description of a considerable release of inosine and hypoxanthine from the heart during ischemia/hypoxia [95]. This study was exclusively focused on the recently identified vasodilatory effects of adenosine, which may act as a feedback mechanism to control coronary flow in situations where the heart works under hypoxic or ischemic conditions. These by-products of ATP breakdown were proposed as possible indicators for myocardial ischemia. Since then, several researchers have verified this intriguing phenomenon—the cardiac efflux of adenosine, inosine, and hypoxanthine- in a variety of animal models of myocardial ischemia [96]. It was proposed that during a lack of oxygen, nucleotides are released from the cardiomyocyte into the interstitium. Then, in an endothelial cell, a rapid catabolism of adenosine occurs, producing inosine and hypoxanthine, which are washed away in the coronary lumen. In the Langendorff perfusion experiments, inosine was determined in the perfusates during normoxia. Inosine release during anoxia was less significant than the release of adenosine. The most dramatic increase in the concentration of inosine was determined after 32 min of ischemia [97]. In 1981, Jennings et al. employed healthy mongrel dogs with in vivo myocardial ischemia and ex vivo heart excision models [98]. Evaluations were conducted on the eluted nucleotides and their by-products, such as hypoxanthine and inosine. They reported that as early as 15 min after the in vivo ischemia and 60 min after the ex vivo global ischemia, a significant elevation of inosine and hypoxanthine concentrations was found in blood or coronary effluent during both ex vivo global cardiac ischemia and in vivo regional ischemia (via ligation of the coronary artery). Other research that used isolated rat or rabbit hearts has revealed similar results [97]. In the other Langendorff perfusion experiments, inosine had a protective effect on cardiac ATP and phosphocreatine concentration during hypoxia and ATP and glycogen content during low-flow ischemia [31,99]. It was found that inosine was able to enter the underperfused rat myocardium. At the beginning of global ischemia, inosine attenuated the breakdown of ATP, but on the other hand, at the end of the ischemic period, the ATP concentration was similar whether or not inosine had been administered [100]. Schneider et al. found that during postischemic reperfusion, inosine, in a dose-dependent manner, normalised coronary perfusion and increased ATP content at the highest dose applied. At higher doses, it increased adenine nucleotide synthesis through hypoxanthine by the salvage pathway [101]. When given every 30 min, 4 mM inosine was reported by Duvall-Arnold et al. to enhance ATP levels in isolated working rat hearts subjected to a 2-h ischemic cardioplegia. Additionally, after 30 min of reperfusion, the ATP recovery showed a slight improvement. Inosine could have further enhanced the ATP regeneration and functional recovery if it had been added to the perfusion fluid. Eventually, it was discovered that, in dogs undergoing normothermic global ischemia and cardiopulmonary bypass, EHNA blockage of adenosine deamination and inosine infusion (500 µg/min/kg) improved the preservation of ATP concentrations at the end of ischemia and recovery compared to when EHNA or inosine were given alone [53].

Remarkably, de Jong and colleagues showed that there were significant age-related variations in purine release in isolated adult or neonatal rat hearts [102,103]. Adult hearts released 58% of their inosine and 18% of their adenosine during the early stages of reperfusion, while neonatal hearts released 38% of their inosine and 53% of their hypoxanthine. An enzymatic experiment supported the authors’ interpretation of the data, which suggested that newborn hearts had reduced xanthine oxidoreductase (XOR) activity. In those hearts, ATP-catabolite release following reperfusion was decreased in comparison to the adult hearts, and this was accompanied by inhibited xanthine oxidase activity. In the modulation of nucleotide metabolism in ischemia and reperfusion injury, superoxide anion derived from xanthine oxidase was a contributor to post-ischemic cardiac injury, which occurred right after the reperfusion. Inosine release plays an important role in the production of superoxide anion at the time of initial reperfusion, while adenosine release has no effect in this condition [104]. It was found that inosine may have a more direct impact on intracellular signalling compared to adenosine, which mostly operates through surface purine receptors. It was proposed that by disrupting the PARP activation pathway, inosine can potentially have cytoprotective benefits. This presumption was supported by the structural resemblance between inosine and NAD+, the PARP substrate. The study of Szabo et al. determined whether inosine affects the PARP pathway during ischemia/reperfusion and how it affects ischemia/reperfusion injury. The heterotopic heart transplantation model was used to replicate clinical settings for whole blood reperfusion and to enable a 24-h observation period—something that was not feasible with isolated organ models. Moreover, the heterotopic condition makes it possible to evaluate cardiac function apart from the real loading circumstances. It was shown that global profound hypothermic ischemia for heart preservation, followed by reperfusion, significantly activates PARP, which inosine suppresses [54]. In the other experiments, ischemic hearts were perfused with Krebs buffer enriched with salicylic acid, a common anti-arthritic or anti-inflammatory drug used in modern clinical practice. At a concentration of 1.0 mM, inosine efflux was roughly nine times higher than in hearts without salicylic acid. The enzyme that converts inosine to hypoxanthine, purine nucleoside phosphorylase, was not significantly inhibited by salicylic acid (0.1 or 1.0 mM), indicating that the increased inosine efflux was caused by the salicylic acid’s action on upstream components of cellular respiration. Despite a considerable increase in inosine efflux (2.7-fold), perfusion with 0.1 mM salicylic acid resulted in a substantial functional improvement, while 1.0 mM salicylic acid further reduced postischemic cardiac function. It was determined that salicylic acid-induced effects on cellular respiration and myocardial ischemia may be detected with high precision using inosine as a biomarker. Interestingly, in this ex vivo model, the inosine efflux concentration was not a good indicator of each patient’s post-ischemic heart functional recovery [105]. In isolated guinea pig heart perfusion, it has been noticed that EHNA influenced purine metabolism. It not only increased post-ischemic ATP and phosphocreatine restoration but also lowered tissue inosine and IMP concentration. During reperfusion, adenosine metabolism can be controlled to produce superoxide anion, which is crucial for the cardiac recovery of ischemic myocardium. Only the release of inosine during the initial reperfusion is linked to the formation of superoxide anion [106].

Moreover, inosine is a positive inotropic drug that dilates the coronary blood arteries during ischemia. An infusion of inosine lowers blood pressure, which reduces cardiac injury. Inosine improved the isolated perfused working heart’s function both under control and during the postischemic reperfusion phase by dose-dependently increasing coronary flow. When ischemia occurs, inosine prevents the loss of ATP and enhances functional recovery after reperfusion. Even with the highest doses of inosine, cardiac output (CO) was lower than the preischemic control [101]. Inosine-infused rat heart preparations showed increased left ventricular systolic pressure and CO after reperfusion [53]. Inosine-treated rabbit hearts improved contractile function, as measured by dp/dt. The postischemic improvement in cardiac function could be related to the primary increase in the coronary flow, as in the control normoxic state [101]. In patients with chest pain examined at the emergency room, the concentration of inosine was usually found to be low or non-detectable. This could be attributed to the length of time the whole blood sample was left to separate the heparinised plasma from the blood cells. Red blood cells (RBCs) originate from human whole blood and contain the enzyme purine nucleoside phosphorylase (PNP), which converts inosine into hypoxanthine. In an earlier investigation using ex vivo Langendorff perfused mouse hearts perfused with an RBC-free Krebs buffer solution, significant levels of inosine and extremely low to undetectable concentrations of hypoxanthine were observed after 20 min of global myocardial ischemia. The relative increases in inosine and/or hypoxanthine among these ex vivo and in vivo experiments would depend on the presence or absence of PNP. Since a higher release of protein biomarkers indicative of acute myocardial ischemia tissue necrosis is typically detected many hours after the occurrence of an AMI, an elevated inosine level should potentially appear in the bloodstream before any increase in these biomarkers [107].

### 4.5. Hypoxanthine

The pioneering study performed by physiologist Robert M. Berne in 1963 contains the first report that the heart is capable of releasing appreciable concentrations of inosine and hypoxanthine during ischemia/hypoxia [95]. Hypoxanthine is either recovered for the ATP pool by hypoxanthine-guanine phosphoribosyl transferase (HGPRT) or oxidised by XOR, generating harmful oxygen species in the reoxygenated hypoxic heart [108]. A study by Oliveira et al. aimed to clarify the role of carvedilol in preserving cardiac mitochondria from oxidative damage caused by hypoxanthine/xanthine oxidase, a recognised source of oxidative stress within the vascular system. The data demonstrated that carvedilol partially safeguarded cardiac mitochondria from damage caused by oxidative stress [109]. Harmsen et al. discovered the possibility of incorporating purines into the myocardial GTP pool. The purine incorporation rate was about 25% of that of ATP. Noteworthy is that the heart’s GTP content is only 5% of the ATP concentration. In the heart, hypoxanthine and inosine can enter the ATP and GTP pools. The process of incorporation is stimulated after ischemia and ribose perfusion. It is dependent on the cardiac PRPP level. According to these findings, it was found that inosine is a better nucleotide precursor to restore ATP levels than hypoxanthine [31]. Hypoxanthine is a tiny polar compound with a low molecular weight of 136 Da. As a result, it can be quickly transferred into the bloodstream through passive diffusion from damaged cardiac tissue. Researchers have validated this intriguing phenomenon—the cardiac efflux of hypoxanthine—in several animal models of myocardial ischemia. For instance, Jennings et al., in 1981, employed healthy mongrel dogs that experienced both in vivo (heart excision) and ex vivo myocardial ischemia [98]. Evaluations were conducted on the eluted nucleotides and their by-products, such as hypoxanthine and inosine. They reported that as early as 15 min after the in vivo ischemia and 60 min after the ex vivo global ischemia, a significant elevation of inosine and hypoxanthine concentrations was found in blood or coronary effluent during both ex vivo global cardiac ischemia and in vivo regional ischemia (via ligation of the coronary artery) [110]. Other research that used isolated rat or rabbit hearts has revealed similar results [111,112]. It indicated that there were significant age-related variations in purine release in isolated adult or neonatal rat hearts. Adult hearts released 58% of their inosine and 18% of their adenosine during the early stages of reperfusion, while neonatal hearts released 38% of their inosine and 53% of their hypoxanthine. The hearts of newborns released less ATP-catabolite upon reperfusion than the hearts of adults, and this was accompanied by a decrease in xanthine oxidase activity [102]. Another ex vivo study using Langendorff perfused mouse hearts was performed using a well-established protocol of cardiac ischemia/reperfusion. A significant amount of hypoxanthine (>7 fold) was found in the perfusate originating from the mouse hearts following 20 min of global ischemia, as compared with the non-ischemic normoxic control hearts [55]. In the other experiments, to characterise the repartition of hypoxanthine between the two pathways, rat hearts were exposed to hypoxia for 20 min, and the recovery (ventricular, end-diastolic, and coronary pressures, as well as the contraction rate measurements) was observed during the 30-min reoxygenation period in the presence of either hypoxanthine or guanine alone, or both. In hearts that were reoxygenated with 100 μm hypoxanthine and 100 μm guanine, the rate-pressure product regained 78% of the pre-hypoxia values; in contrast, 49% of the pre-hypoxia values were restored in the presence of both hypoxanthine and guanine (100 μm each). As guanine competes with hypoxanthine in the same location of HGPRT, hypoxanthine is likely spared when it is present alone and is oxidised, causing the reperfusion injury. When xanthine was substituted for hypoxanthine, the functional impairment decreased more slowly and was removed by catalase and superoxide dismutase, suggesting that hazardous oxygen species produced by XOR and hypoxanthine are the source of the injury. These findings imply that the salvage pathway might play a key role in shielding hypoxic hearts from reperfusion damage [113].

Clinical practice analyses of human plasma using HPLC-UV suggested that inosine and hypoxanthine were possible biomarkers for acute myocardial ischemia. For instance, cardiac hypoxanthine release was demonstrated in the serum of patients undergoing an atrial pacing stress test with documented angiographically ischemic heart disease [59]. A proven quantitative HPLC-UV method was also utilised to gather and assess residual plasma samples (heparinised) from patients experiencing non-traumatic chest pain in the emergency room. Hypoxanthine concentrations were found to be significantly elevated compared with those in the control subjects [107]. The concentrations of total inosine and hypoxanthine are found endogenously in the body and metabolised in the normal purine cycle in almost all cell types and organs; therefore, the specificity of these two biomarker candidates of acute cardiac ischemia is relatively low, in contrast to those of highly specific biomarkers such as troponins. The extraordinarily high sensitivity of inosine/hypoxanthine for identifying the early beginning of myocardial ischemia, however, may outweigh its limited cardiac specificity drawback. Chemiluminescence technology is one of the most accessible and sensitive analytical techniques currently used. The published results showed a quick, sensitive, and highly repeatable way to measure plasma total hypoxanthine concentrations in healthy individuals versus cardiac patients with confirmed cTnT elevation [60].

## 5. Conclusions/Summary

This publication focuses on the role of purine nucleotide precursors, D-ribose, AICAR, inosine, adenine, and hypoxanthine, in the processes of myocardial ischemia and reperfusion. Despite decades of progress in cardiovascular medical technologies, cardiovascular disease remains the world’s leading cause of mortality for both men and women [114]. One of the main causes of death for middle-aged and older people has remained myocardial ischemia, a component of cardiovascular disease [115]. Improvements in nutrition, regular exercise, smoking cessation, and blood pressure control are just a few of the cardiovascular risk factors in this condition that have been addressed with ongoing, extensive education. Still, more research is needed to fully understand the underlying metabolic energy deficit associated with myocardial ischemia. Morbidity and mortality from myocardial ischemia and related acute coronary syndromes remain extremely high despite the above treatment approaches [116]. This has led to an intense search for additional “innovative” therapies to treat or prevent myocardial ischemia and the associated tissue injury. For many years, the focus of research has been on creating methods for restoring low ATP levels both during and after myocardial ischemia. Many studies have been conducted on providing metabolic substrates for the regeneration of ATP molecules after ischemia. During ischemia, the levels of these precursors can fluctuate, reflecting metabolic stress and cellular damage. Upon restoration of blood flow—that is, during reperfusion—there are rapid changes in nucleotide metabolism, which can lead to the formation of harmful products such as uric acid from hypoxanthine. These processes are associated with oxidative stress and cell injury, impacting the overall condition of the heart muscle. The publication emphasises that monitoring the levels of purine nucleotide precursors can help assess the extent of heart damage and the effectiveness of reperfusion therapy. Additionally, the authors suggest that manipulating the metabolism of these compounds could serve as a potential therapeutic target aimed at minimising damage and improving heart function after episodes of ischemia and reperfusion. In summary, purine nucleotide precursors play a key role in the pathophysiology of myocardial ischemia and reperfusion, and their study may contribute to a better understanding of the mechanisms of heart injury and the development of new therapeutic strategies to reduce damage and enhance cardiac function following ischemic episodes.

## Data Availability

No new data were created or analyzed in this study. Data sharing is not applicable to this article.
